# Resilience among asylum seekers living with HIV

**DOI:** 10.1186/1471-2458-12-926

**Published:** 2012-10-30

**Authors:** Lois Orton, Jane Griffiths, Maia Green, Heather Waterman

**Affiliations:** 1 Division of Public Health & Policy, Institute of Psychology, Health & Society, The University of Liverpool, 3rd Floor, Whelan Building, 1-5 Brownlow Street, Liverpool, L69 3GL, UK; 2 The School of Nursing, Midwifery and Social Work, The University of Manchester, Room 6.337, Jean McFarlane Building, University Place Oxford Road, Manchester, M13 9PL, UK; 3 Social Anthropology, The University of Manchester, Arthur Lewis Building, Oxford Road, Manchester, UK; 4 The School of Nursing, Midwifery and Social Work, The University of Manchester, Room 6.314a, Jean McFarlane Building, University Place Oxford Road, Manchester, M13 9PL, UK

**Keywords:** UK, Asylum seeker, HIV, Stress, Resilience

## Abstract

**Background:**

A small body of evidence demonstrates the challenges faced by migrant communities living with HIV but has yet to consider in-depth the experience of asylum seekers whose residency status is undetermined. The overall aim of our study was to explore the experiences of those who are both living with HIV and seeking asylum. This paper focuses on the stressors precipitated by the HIV diagnosis and by going through the asylum system; as well as participants’ resilience in responding to these stressors and the consequences for their health and wellbeing.

**Methods:**

We conducted an ethnographic study. Fieldwork took place in the UK between 2008–2009 and included: 350 hours of observation at voluntary services providing support to black and minority ethnic groups living with HIV; 29 interviews and four focus group discussions with those who were seeking asylum and living with HIV; and 15 interviews with their health and social care providers. Data were analysed using the constant comparative method.

**Results:**

There were three main stressors that threatened participants’ resilience. First, migration caused them to leave behind many resources (including social support). Second, stigmatising attitudes led their HIV diagnosis to be a taboo subject furthering their isolation. Third, they found themselves trapped in the asylum system, unable to influence the outcome of their case and reliant on HIV treatment to stay alive. Participants were, however, very resourceful in dealing with these experiences. Resilience processes included: staying busy, drawing on personal faith, and the support received through HIV care providers and voluntary organisations. Even so, their isolated existence meant participants had limited access to social resources, and their treatment in the asylum system had a profound impact on perceived health and wellbeing.

**Conclusions:**

Asylum seekers living with HIV in the UK show immense resilience. However, their isolation means they are often unable to deal with their treatment in the asylum system, with negative consequences for their perceived health and wellbeing.

## Background

Migrants have come to comprise an increasing proportion of the UK population (from 8% in 2001 to 12% in 2010). The number seeking asylum has fluctuated since 1990, reaching its peak in 2001/2002 (when there were 84,130 applications) then falling until 2010, when there were just 17,790 applications [1]. However, despite new procedures to cut processing time, the numbers of asylum seekers awaiting a decision on their case has actually risen in recent years. This is thought to be largely due to a large backlog of cases from the early 2000’s. As a result, many applicants still find themselves waiting several years for a decision on their case. In the end, the vast majority of asylum applications are unsuccessful (74% of initial decisions in 2010 were refusals). However, it is thought that only a small proportion of those refused asylum are forcibly or voluntarily removed from the UK [1].

There is little routine recording of asylum status among health services. Consequently, it is unclear how many asylum seekers are living with HIV [2]. There are some data for ethnicity. This reveals that 65% of new HIV diagnoses between 2001 and 2010, with country of birth known, were among those born abroad [3] and physicians have reported that many people newly diagnosed with HIV have arrived in the UK relatively recently [4]. Asylum seekers are considered particularly vulnerable to HIV for three main reasons: they may have experienced situations of risk in areas of high HIV prevalence [5]; their migration may have been triggered by such experiences as detention, beatings, torture, rape, sexual assault and harassment [6]; and the experience of becoming an asylum seeker or refugee may involve poor living conditions, malnutrition, lack of protection and depression which may leave them vulnerable to sexual exploitation [7]. Female asylum seekers are hardest hit as a result of gender inequalities.

For those living with HIV and seeking asylum there are many challenges to be faced. Both migrating to a new country and experiencing life as an asylum seeker lead to new experiences that could be interpreted as “stressors” (adverse events that lead to negative outcomes [8]). In the past, researchers tended to document the events (such as war, torture, murder of loved ones and rape) that might have caused asylum seekers to flee [9,10]. These were termed “pre-migratory stressors”. More recent studies have explored experiences in the country where they seek refuge, including: fears of being sent home; problems in accessing health care services; interviews with immigration officials; separation from family; threats to family; poverty; detention; under-employment; loneliness; isolation and discrimination (for example: [11-16]). In the UK, these “post-migratory” stressors are often experienced over an extended period as, despite new immigration procedures (including the New Asylum Model), the backlog of cases means that asylum seekers often wait several years for a decision on their case. In these situations, the effects of pre- and post-migratory stressors are believed to compound one another, with post-migratory stressors, experienced in the country where refuge is sought, having the most damaging impact on mental health [11-13,17], physical health [14] and quality of life [16] overall.

Being diagnosed with HIV is also likely to precipitate new stressors. However, the unique experience for those who are in the process of seeking asylum and for whom their residency status is undetermined remains unexplored. Most research among migrant communities living with HIV is designed to explore access to health care [18-20] and the use of HIV treatments [21-24]. These studies have revealed some stressors, and demonstrate a range of additional challenges for this group, including: coming to terms with the experience of HIV elsewhere as a “terminal illness”; the fear of withdrawal of treatment; and the stigma that precludes contact with home communities and hinders the development of new relationships [24,25]. Previous research has demonstrated that those whose life trajectories show an accumulation of adverse events and a lack of good experiences may be particularly vulnerable to ill health [26]. Thus, it seems likely that the stressors faced in living with HIV would compound pre- and post-migratory stressors and add to negative health outcomes.

However, other research with disadvantaged groups has shown that different people respond in different ways to the same experiences, with some better able to “beat the odds” [27,28]. For example, in their study of poor households in Britain, Canvin et al. revealed many “tales of the unexpected” among participants who were able to cope with very difficult situations contrary to their own, and other people’s, expectations. Current policy in the UK seeks to build on these capacities. In fact, the empowerment of individuals to take control of their own health and wellbeing is now stated as a key aim of many new public policies (for example: [29-33]). On the other hand, current and planned strategies affecting asylum seekers, and particularly those living with a long term condition such as HIV, seem to be moving in the other direction; thereby reducing the opportunity for this group to take control of their health and wellbeing (for example: by dispersing asylum seekers across the UK, disallowing them from working, and preventing access to health care for some groups). Thus, it is important to highlight the difficulties faced by asylum seekers, particularly those living with a long term condition such as HIV, but we must not forget to also explore their capacities in terms of how they respond to these experiences [34,35].

The data presented in this paper derive from a doctoral study exploring the experience of life as an asylum seeker living with HIV. Initially, we were interested in the stressors precipitated by both of these experiences (HIV and asylum) and the ways in which participants responded to them. Whilst participants’ experiences in the asylum system were felt to be detrimental for their health, they also described how they were able to deal with some very difficult circumstances. Here, we present these findings about the resilience processes of people seeking asylum and living with HIV. Resilience is a concept which has often been invoked to describe this ability to react positively when things go wrong. It is a complex phenomenon. Luthar et al. ( [8], p543) developed a comprehensive definition after a critical review of the existing research. They characterise it as “a dynamic process encompassing positive adaptation within the context of significant adversity”. In their definition, resilience is not considered a personality trait. In other words, a person cannot be defined as resilient. Rather, their resilience in any situation depends on the nature of the “stressor(s)” faced and their ability to react positively.

In this paper, the concept of resilience has been invoked to explore: firstly, the combination of stressors that are most important for asylum seekers living with HIV; secondly, how they respond to these stressors including the processes that help them to cope; and, thirdly, the limits of this resilience and the consequences for their health and wellbeing.

## Methods

### Study design and setting

As this study was the first to explore the experiences of asylum seekers living with HIV, an ethnographic approach, based in symbolic interactionist assumptions, was taken to allow previously unexplored understandings and values to be examined [36]. Multiple data generation techniques were used to check early findings and to build a picture that is richly embedded in the research setting. A steering group was convened to support study design and execution and data analysis. This group consisted of academics, HIV and asylum service providers and study participants (asylum seekers living with HIV). Fieldwork took place in the UK between January 2008 and March 2009. The study was restricted to a small number of sites. This helped facilitate an in depth investigation and allowed the establishment of strong links which proved important for the participation of this vulnerable population. HIV support groups that targeted services at the black and minority ethnic (BME) community (including many asylum seekers) were deemed the most appropriate place to begin fieldwork. Later methods involved accessing the two main regional specialist HIV hospital services. Semi-structured interviews and focus group discussions were the main methods of data collection. However, the approach taken in using these methods, and the interpretation of data generated through them, was significantly informed by in-depth observational field notes. Thus, observation began before other methods and continued throughout the period of fieldwork.

#### Sampling and recruitment

By assuming the role of volunteer, the main researcher (LO) began the study by observing support groups run by the two main voluntary HIV organisations targeting the local BME community. The aim of observation was to understand the context of the lives of those seeking asylum and living with HIV, and to explore which issues were most important for them. Consent was sought verbally, at the start of support group sessions, and at frequent intervals thereafter.

As the main issues for service users began to emerge, LO invited those attending the support groups to take part in interviews so they could discuss their experiences in more detail and to explore the aspects of their lives it had not been possible to observe. Potential participants must be adults who identified themselves as seeking asylum at first contact (refugee status was later granted for some), and as living with HIV. They could not be included in the study if they spoke a language for which an interpreter and written study information was unavailable; or if they were severely ill, hospitalised or had a severe mental impairment. During support group sessions, LO presented her research and asked those who were interested in taking part to approach her for more information. Respondents were then provided with an information sheet and a time was arranged for them to meet with LO. As the research progressed, further participants were sought based on the developing analysis (theoretical sampling: [37]). We became interested to learn about the experiences of those who do not attend HIV support groups, to determine if there is any difference in their resilience processes. Thus, subsequent participants were sought from both the support groups and NHS HIV clinics. LO explained to staff at both sites about what characteristics she sought in study participants. These staff then identified people who met these criteria and provided them with an information sheet. Those who were interested were invited to speak with LO, who was sat in a nearby private room. Early data analysis revealed voluntary and NHS service providers to be an important source of support for study participants. Consequently, they were approached directly by LO, provided with an information sheet and asked to get in touch if they wished to take part in an interview.

Once most interviews were complete, two male-only and two female-only focus group discussions were conducted with “natural” groups of participants recruited from the support groups, in order to further explore and test the main themes emerging from the analysis. The aim was not to reach the “truth” but to check whether the research account was recognisable for participants, and to determine what was missing from the analysis. It also helped to enhance the relevance of the research to participants [38]. A general invitation was made at the support groups. Participants informed LO or their service provider if they wished to take part and were provided with an information sheet including the time and place of the focus group sessions.

### Data generation

Three hundred and fifty hours were spent observing and taking part in HIV support groups. LO recorded the context of participants’ lives through detailed field notes. These notes included direct quotes and observations as well as feelings and responses to the situations encountered. On initial entry into the field the aim was to observe and record every part of the support group context and make broad and detailed descriptive observations, what Spradley ( [39], p55) terms “grand tour observations”. As time progressed the observation became increasingly focussed on those aspects that appeared most appropriate to the emerging analysis, and to themes that had arisen from other concurrent data collection processes. LO began to ask specific questions of research participants and to focus her note-taking on those aspects relating most directly to the emerging framework of analysis.

Formal interviews were a natural progression from this informal questioning. Twenty-nine semi-structured interviews were conducted with 26 asylum seekers living with HIV, including three follow-up interviews six months after the first interview. Interviews began with a discussion of the study and what participation would involve. After written informed consent was taken, a schedule was then used to explore the most important aspects of participants’ lives and how they dealt with the experiences they faced. The schedule was not used rigidly. Instead it served as a guide to topics and an aid to question-asking. Participants were allowed to largely direct the interview in order to explore the issues of importance to them. One participant did not feel comfortable conversing in English, so a trained interpreter (recruited from the HIV voluntary organisations) was used to help explain the study, take consent and conduct the interview. This interpreter was also interviewed to gain their perspective on the topic, and to explore how this may have affected the interpretation process [40]. Five interviews were conducted with voluntary organisation staff; and 10 with NHS staff. A brief schedule was used in these interviews to explore their experiences in caring for asylum seekers and the support they provided. All interviews lasted between 45 minutes and an hour and a half. The four focus groups included between six and 12 participants and lasted between an hour and a half and two and a half hours. After taking written informed consent the discussion, guided by LO and a trained peer co-facilitator, was structured around the emerging findings. Interviews and focus group discussions were recorded electronically and were transcribed verbatim. For the interview in which an interpreter was used, everything was transcribed (in both languages).

### Data analysis

Analysis occurred concurrently with data generation using the constant comparative method [31]. The process of interpretation was critical of the values, ideas and presumptions that the researcher has brought to the research as a co-participant in each encounter (reflexivity). Field notes and transcripts were entered into the software package NVivo 8 and were coded line-by-line based on the meanings, perspectives, and actions they represented, and for contextual factors in their generation. The aim was to identify themes grounded in the data ( [41]: p32); and to convert them into an explanation of the situation that had resonance with relevant groups and that could convince others of its plausibility [42]. There became a focus on participants’ abilities to deal with the stressors that were precipitated by the asylum system and compounded by a HIV diagnosis. This paper describes participants’ resilience, from a social constructionist perspective.

### Ethical considerations

This study, as much ethnographic work, involved the less powerful. The main researcher (LO) was a white, British, female researcher and PhD student. Most participants, on the other hand, were recent black African immigrants living in relative poverty, seeking asylum and full entry to the UK. Despite many being of relatively high social standing in their country of origin, they had experienced a drop in status since becoming an asylum seeker and were vulnerable due to their HIV status.

In order to avoid exploitation, LO was mindful to act in a sensitive, critical and reflexive manner throughout the research process [43] to establish maintain and nurture “reciprocal and respectful relationships” with study participants ( [44], p97). Her behaviour was guided by her long term engagement at the research site. However, she could not assume that by taking this sensitive approach she could automatically avoid all pitfalls [45]. Ethical issues were discussed at length with supervisors, gatekeepers and the steering group before the study began. Research methods were designed to minimise the chance of coercion or exploitation and protocols were set-up to flag and resolve any potentially damaging consequences. Extra time was set aside to ensure that participants were aware of what taking part in the study would entail, as well as the limitations of the study, before consent was taken to participate, and throughout participation in the study. These methods were approved by the University of Manchester Research Ethics Committee and Central Manchester Research Ethics Committee. Ethics approval disallowed the use of direct quotes from observational material due to the impossibility of taking written informed consent in an ever-changing environment – the support group. However, as outlined above, observational data has been used to develop the research question, to inform interview and focus group methods and to identify the key themes presented in this paper.

## Results

Table 1 provides a summary of key demographic data for the main study participants – those who were seeking asylum and living with HIV. Excerpts are indicated in the text by the code PI. These participants were aged between 25 and 45 years. Slightly more women than men took part. Most had children (who often remained in their country or origin). About half were from Zimbabwe. The rest were from a wide range of (mainly African) countries. Most had sought asylum 5–6 years ago. The vast majority were diagnosed with HIV after they sought asylum.

**Table 1:** Key demographic data for asylum seekers living with HIV taking part in an interview

**Age range**	**25** – **45 years**
**Gender distribution**	Female (16)
	Male (10)
**Frequency of participants with dependents**	Has dependents (20)
	No dependents (2)
	Undisclosed (4)
**Country of origin**	Zimbabwe (13); Malawi (4); Nigeria (3); Rwanda (1); Lesotho (1); Brazil (1); South Africa (1); Uganda (1); Cameroon (1)
**Immigration status** (**Home Office definition**)	Asylum seeker (17)
	Asylum seeker in appeal (8)
	Granted limited leave to remain (1)
**Year of asylum application**	1998 (1); 2001 (1); 2002 (7); 2003 (8); 2004 (4); 2005 (2); 2006 (2); 2008 (1)
**Year of diagnosis**	1996 (1); 1997 (1); 1998 (1); 2000 (1); 2001 (2); 2002 (4): 2003 (5); 2004 (6); 2005 (3); 2006 (1); 2008 (1)

Please note, data have been aggregated to protect the identity of individual research participants.

Voluntary organisation staff taking part in this study were involved in running the support groups and had regular contact with service users who were seeking asylum. Excerpts are indicated in the text by the code VS. NHS staff (indicated with the code NS) had a variety of roles. Below is a breakdown of self-defined professions.

•Specialist midwife in HIV.

•HIV community liaison nurses.

•nurse registrar.

•HIV counsellor.

•HIV clinical psychologist.

•HIV consultants.

•1 specialist nurse in HIV treatment adherence

### Seeking asylum and living with HIV: a unique combination of stressors

A wide range of stressors, related to both seeking asylum and living with HIV, were experienced by those taking part in our study, including:

•Living in limbo unable to create a future or to go back to their old way of life

•Not being allowed to work leading to feelings of uselessness, redundancy, loss of status, being deskilled and unable to provide for themselves and their family

•Living in poverty

•Loss of family life and community

•Exclusion from the process of influencing the outcome of one’s asylum case

•The shock of the HIV diagnosis and the fear of death

•Experiencing internal and external stigma

•The burden of HIV treatment

Amongst these, there appeared to be three main threats to participants’ resilience. First, they had left behind many resilience resources as a result of the “cataclysmic stressor” [46] of migration. This included the loss of strong social ties, a rewarding job role, and a stable identity and social and cultural structure. Second, stigmatising attitudes towards HIV were very common among participants’ home communities. Despite most being diagnosed in the UK, home stigma still had a profound social impact. Their condition became a taboo subject that could not be discussed with friends and family both at home and also in the UK. This contributed to their sense of isolation. Third, participants found themselves trapped in limbo, unable to influence the outcome of their asylum case, and reliant on HIV treatment to stay alive. HIV treatment was very important to participants. Experience told them that in the countries from which they had fled drugs options were highly limited, inaccessible, unaffordable, in short and inconsistent supply. Furthermore, the criteria for starting treatment often required that only the sick and dying were eligible. Treatment made life with HIV feel manageable. However, if they were refused asylum they once more faced their worst fear, of HIV as an unmanageable condition, leading to inevitable death. Many likened being returned to their home country to a “death sentence”. This can be juxtaposed against life as an asylum seeker living with HIV and receiving treatment, which was described by one participant as a “life sentence” (PI07, male). Thus, whilst their original reasons for applying for asylum usually had nothing to do with HIV, or “health tourism” [47], their diagnosis became an extra reason to fight to stay in the UK. As a result, participants remained trapped in limbo in the asylum system, unable to work, away from their family and community, experiencing poverty and fearing deportation.

Participants described how the stress of being trapped in limbo could lead to feelings of anxiety, panic and depression. Some felt this stress had directly affected their physiology, causing their CD4 count (a marker of immunity used to measure suppression of the HIV virus) to drop:

"… you end up seeing that your CD4 is dropping down, because CD4 deals with your emotions. You can be taking your medication every time, but, so the fact that you are depressed, you are stressed, your CD4 goes down… your consultant says, have you, have you missed any medication? No. But why your CD4- ? Oh I’ve got this and this and - OK, we know that you are not settled, so this immigration problem, cause you are living in worry, you don’t know what will happen with you tomorrow, you don’t know whether your children will be withdrawn from the school and then you have to pack and go."

(FGD03, male participant)

### Resilience processes

In order to understand participants’ resilience, from a social constructionist perspective, it matters how they interpreted their own abilities to cope with these stressors [48]. Most drew on a range of resilience resources. Some took part in voluntary work, others enrolled in educational or training courses, became involved in health promotion activities, or campaigned for asylum seekers’ rights. Recognition of individual’s strengths and capabilities has previously been shown to encourage positive planning for the future [27]. In our study, staying busy helped many participants to avoid dwelling on their situation, and allowed them to increase their skills and employability, giving meaning and purpose to life in limbo:

"…[I] put my name down to go to French and for training - trying to keep myself busy, you know. And having to be looking forward to doing things, maybe make me feel excited."

(PI03, female)

For others, their faith was an important resource. In order to stay strong they needed to accept what was happening to them. The UK government did not seem to take decisions in their best interest, and they struggled with their lack of control over the outcome of their case. By turning to God, they could focus their efforts towards someone who was believed to be looking out for them:

"’cause nobody has the power it seems…but it’s only the government who has… so I just keep every day you are praying, you know, even if you are not a ‘Prayer’, but in this country with this asylum process, you end up being the strongest person you can ever think of, the only person you know to help you is God. And it’s only God that will, you just say ‘God will you take control of my things’…"

(PI10, female)

This highlights the importance, when faced with a lack of agency, of relocating power to a source which is felt to be legitimate [49]. Health and social care staff from NHS and voluntary sites also became important figures in helping participants to see a future with HIV. HIV consultants were the centre of care received at hospital sites. They were described by participants as non-judgemental and welcoming and were held in high esteem. Many developed a close and trusting relationship. They would discuss personal issues, not just about their health, but relating to all aspects of their lives. They acted akin to friends and provided the social and emotional support known to be important for positive adaptation [27,46]. For many taking part in our study, the interest shown by these high status professionals helped enhance their sense of self worth and the meaningfulness of life in limbo:

…I feel I can say anything to him. I will walk in, the minute I walk in he'll notice, oh you've got, you've changed your hairstyle. And I say, oh, you know, you know what? You always seem to notice something about me, you know, always, always. He's so nice… He even said I was a living miracle cos I mean, the fact that I was in a coma and then all of a sudden I was up and walking…

(FGD04, female participant)

Those who were able to attend support groups also drew confidence from witnessing others surviving and living well. With time, most came to accept their diagnosis and to place less importance on it:

"I accepted it as it is because I never went to buy it, and nobody wants to be, you understand, but when you find yourself in this situation, take it as it is and move forward, and this is exactly what I'm doing."

(PI07, male)

This helped them process their self-stigmatisation and to stabilise their personal identity. However, the majority of participants felt unable to predict or manage the reactions of others (external stigma; [50]). In some cases previous bad experiences had led them to avoid social situations where disclosure might become an issue. Often, this meant the end of close relationships:

"Me, I just understand, because I'm sick…people think bad for that… Me I don't know what to say, how can I find somebody to understand me, I just stay quiet and no speak about that you know. This is why sometimes I am, I have difficult to have friends…"

(PI19, female)

Furthermore, many would shun social activities so their pills could be taken out of view. Participants made a particular effort to avoid contact with people from their home country, in order to keep their diagnosis secret from those they had left behind:

"I don't have any relationship with people from my country… I don't mind other people knowing that I'm positive, I'm not bothered if Zimbabweans come to my house and they find out, that is their business, but I don't want my people from my country to know that I'm positive."

(FGD04, female participant)

Fears of external stigma prevented some from accessing HIV support services and, for those who did, it limited their social interactions at these sites. Thus, as previous researchers have found [51], resilience processes employed to deal with external stigma often had the adverse outcome of contributing to self exclusion, avoidance and social withdrawal.

### The limits of resilience

Participants laid great importance on being able to portray themselves as self-sufficient and adept at dealing with life’s challenges:

"That’s very important I know from experience because you’re alone anyway. You really need to be tough upstairs because things bring you down like that - and you’ve got every reason to give up really, you know what I mean? Your case has been refused, you’re living in a horrible place. So mental toughness - is needed…"

(FGD03, male participant)

Indeed, most were able to draw on a number of resources in order to deal with the many stressors faced. However, participants were living a liminal existence, excluded from British society due both to their status as asylum seekers and as a result of their HIV diagnosis. They were in a permanent state of transition, unable to determine their own way of life or to plan for the future.

"…you can't do what you're supposed to do as a human being. You find yourself like you are just locked in a space whereby you can't, you can't go ahead, you can't see ahead, you can't even plan for yourself, you don't know what… you can do tomorrow."

(FGD02, male participant).

Consequently, their ability to acquire new resilience resources was limited. A lack of social resources appeared to be most damaging for their health and wellbeing. Participants had left behind their community, found themselves unable to confide their diagnosis to those at home, and so to receive their support, and faced difficulties in forming new social bonds in the UK due to their situation as both asylum seekers and the stigma surrounding their HIV diagnosis. Support groups were important in overcoming these experiences, but were not enough to replace the loss of family, community, and culture.

"I come from Africa where family is part – we've been strong because of our family being with people around you, supporting you, sharing problems and whatever. And then you find yourself you are alone, you've run out from your country, you are alone. However much you have support from organisations, it's not as when you have support from the people who understand you and accept you. Sometimes you think you are abandoned each and every person."

(PI08-2, male)

As a result, participants were not always able to process the stress associated with their treatment in the asylum system and feared, above all, that they would be refused the right to stay in the UK. One woman, who had previously commented that she felt well, discussed how she had recently developed physical symptoms since her last appeal had been rejected:

"There are things wrong there, which unexplainable. And maybe by the time I see my consultant they have gone. You wake up sometime maybe the legs are so painful, you stretch your arms like, sometimes you feel like you are sore somewhere, or sometimes I have rash, and, or cough, or. You know, I don’t really feel. Sometimes I wake up and I’m very, very tired. I don’t know what, just tired, and I just want to stay in bed. And then sometimes I don’t have appetite."

(PI01, female)

Thus for her, and for the majority of participants, experiences precipitated by the asylum system were perceived as having a direct impact on one's health and wellbeing.

## Discussion

This is the first study to reveal the impact a HIV diagnosis can have when it comes on top of the stressors experienced by those who are seeking asylum. Findings indicate the resourcefulness of those who are seeking asylum and living with HIV. They also suggest the tenuous nature of resilience. Participants were at risk by virtue of having left behind many resilience resources and being unable to find new ones whilst living in transition. In particular, many were experiencing extreme social isolation as a result of migration (and the associated loss of family support and social structure) and the stigma surrounding their diagnosis (which inhibited the formation of new relationships). Consequently, they were not always able to process the stress associated with their experiences in the asylum system, and these were often perceived to have a direct impact on their health and wellbeing. Thus, whilst this study did not originally set out to explore resilience, it reveals the capacities of people who are seeking asylum and living with HIV. It makes no effort to measure “actual resilience” quantitatively, but reveals participants’ experiences and their resilience processes.

One of the aims of this study was to give a voice to a marginalised group. However, the low social position of participants as asylum seekers may have restricted how far this was achieved in practice. The collection of observational field notes may have helped to overcome this difficulty to some extent by allowing more “natural” data to emerge over a long period of time. Further, as research mainly took place in the “safe” setting of the HIV support group, most participants appeared to feel relatively comfortable in discussing sensitive issues. Our research was highly focused. This contributed to its success in revealing in-depth insights from the lives of those who normally remain remote from research. However, it also presented a limitation. As the vast majority of participants were recruited through HIV support groups it is not possible to fully determine how the resilience, and the perceived health, of those who do not attend support groups would differ. Future studies should seek to include those who do not attend HIV support groups in order to determine the extent to which they are able to access resilience resources to deal with the stressors described here. Building on the findings of our study, future research could also explore ways to strengthen the resilience of asylum seekers living with HIV.

However, it is not enough to focus on people’s resilience in order to mitigate the effects of disadvantage or to expect them to pull themselves out of it. The root causes must be the main target for action [27]. Our study highlights the direct impact of public policy relating to asylum seekers on the lives of real people, demonstrating the importance of taking these impacts into account in order to protect this vulnerable group. These findings come at an important juncture as immigration and asylum seekers' rights are high on the political agenda, with a move towards increasingly restrictive strategies. Theoretical critics, ethicists and political analysts have discussed how the liminal position of asylum seekers and their exclusion from country protection has led them not to be awarded the human rights taken for granted by citizens [15,52-55]. With the changing economy, a perceived rise in asylum seeker numbers, and a heightened fear of outsiders, receiving countries have begun to focus on controlling them with conditions of reception often being designed adversely as a “humane deterrence” [56,57]. Furthermore, whilst some UK policies have taken poverty and social exclusion very seriously, certain vulnerable groups (including asylum seekers) are not covered by specific initiatives and policy has in fact increased exclusion and inequality [58]. Extant studies suggest that British legislation towards asylum seekers reduce their control over their own lives, are fundamentally disempowering [10,34], and may have a negative impact on the development of resilience to mental health problems [34].

Our study confirms these concerns. It suggests that, despite their immense resilience, those who are living with a long term condition such as HIV, believe their mental and physical health is affected by current legislation. The move to increasingly restrictive government policy is likely to threaten many of the resilience resources described in this study, and to further raise the importance of post-migratory stressors. In particular, the policy to disperse asylum seekers across the country, to restrict their access to health and social care services and to move HIV care from specialists to general practitioners, may prevent patients from forming the kind of empowering relationships with their health care providers which have emerged as important for our study participants. Our study has shown how HIV voluntary service providers and NHS staff can make a difference by taking a wholistic approach to the care of those who are not just presenting with a health condition but also with complex social care needs. However, the support they can provide will always be limited by the structures within which they work. Thus, whilst our study highlights the strengths and capabilities of individuals who are supported by their care providers; the findings also suggest the need for political action, for reform of the UK asylum system and for changes in the treatment of asylum seekers in the health care system, particularly those living with a long term condition, such as HIV.

## Conclusions

Asylum seekers living with HIV in the UK show immense resilience despite facing a range of stressors as a result of both their HIV status and through experiencing life as an asylum seeker. However, their isolated existence makes it difficult for them to deal with their treatment in the asylum system and this has negative consequences for their perceived health and wellbeing. Furthermore, various resources identified in this study as being important for resilience are currently under threat as a result of increasingly regressive asylum and health care policies.

## Competing interests

The author(s) declare that they have no competing interests.

## Authors' contributions

LO conceived of the study, designed the study, carried out the fieldwork, analysed the data and drafted the manuscript. JG provided feedback on the study design and fieldwork, contributed to the analysis and commented on drafts of the manuscript. MG provided feedback on the study design and fieldwork and commented on the analysis. HW provided feedback on the study design and fieldwork and commented on the analysis and drafts of the manuscript. All authors read and approved the final manuscript.

## Pre-publication history

The pre-publication history for this paper can be accessed here:

http://www.biomedcentral.com/1471-2458/12/926/prepub
